# Elasto-Plastic Mechanical Properties and Failure Mechanism of Innovative Ti-(SiC_f_/Al_3_Ti) Laminated Composites for Sphere-Plane Contact at the Early Stage of Penetration Process

**DOI:** 10.3390/ma11071152

**Published:** 2018-07-06

**Authors:** Jingchuan Liu, Lan Zhang, Fengchun Jiang, Mengqi Zhang, Liquan Wang, Feihong Yun

**Affiliations:** 1 College of Mechanical and Electrical Engineering, Harbin Engineering University, Harbin 150001, China; liujingchuan@hrbeu.edu.cn (J.L.); wangliquan@hrbeu.edu.cn (L.W.); yunfeihong@hrbeu.edu.cn (F.Y.); 2 Key Laboratory of Superlight Materials & Surface Technology, Ministry of Education, College of Materials Science and Chemical Engineering, Harbin Engineering University, Harbin 150001, China; fengchunjiang@hrbeu.edu.cn; 3 College of Mechanical Engineering, Northwestern Polytechnical University, Xi’an 710072, China; mengqi.zhang@mail.nwpu.edu.cn

**Keywords:** continuous SiC fiber, Ti/Al_3_Ti Metal-Intermetallic-Laminate (MIL) composite, microstructure characterization, elasto-PLASTIC mechanical properties, numerical equivalent inclusion method

## Abstract

A novel silicon carbide (SiC) continuous ceramic fiber-reinforced (CCFR) Ti/Al_3_Ti Metal-Intermetallic-Laminate (MIL) composite was fabricated. A high-efficiency semi-analytical model was proposed based on the numerical equivalent inclusion method (NEIM) for analyzing the small-strain elasto-plastic contact in the early stage of the penetration process. The microstructure and interface features were characterized by the scanning electron microscopy (SEM). Quasi-static compression tests were performed to determine the contact response and validate the proposed model. A group of in-depth parametric studies were carried out to quantify the influence of the microstructure. The comparison between results under the sphere-plane and plane-plane contact load indicates that, under the first sphere-plane, the compressive strength and failure strain are both lower and the SiC reinforcement effect on strength is very clear while the effect on ductility is not clear. The maximum plastic strain concentration (MPSC) in the Al_3_Ti layer is closest to the upper boundary of the central SiC fiber and then extends along the depth direction as the load increases, which are also the locations where cracks may initiate and extend. Moreover, the CCFR-MIL composite shows better mechanical properties when the center distance between adjacent SiC fibers is four times the fiber diameter and the volume fraction of Ti is 40%.

## 1. Introduction

### 1.1. Development of Metal-Intermetallic-Laminated Composite

Ti-Al intermetallic compounds have drawn great attention in recent years for their enormous potentials of structural applications in the spacecraft, aviation, and car industries due to their outstanding mechanics performances. This includes their high specific strength and melting point, low density, excellent creep resistance, and inoxidizability at high temperatures [1,2,3]. However, these intermetallic compounds exhibit poor ductility and deformation capacity under a room-temperature condition, which restrict the value of engineering applications [4,5]. Given that, the composite molding technique was adopted to enhance the ductility of intermetallic compounds by introducing the reinforcement elements such as particles [6], fibers [7], ductile metal intergranular phases [8], and layers [9,10,11] into a brittle intermetallic matrix. In particular, through the bionic design, the metal-intermetallic laminated (MIL) composites were successfully fabricated. The metal layer of ductile Ti in an MIL composite was employed as a reinforcement to strengthen the toughness of the whole material [12,13,14]. This bionic design uses the energy dissipation principle and aims to minimize the impact of crack initiation on mechanic performances of the composite material [15].

**Figure 1:**
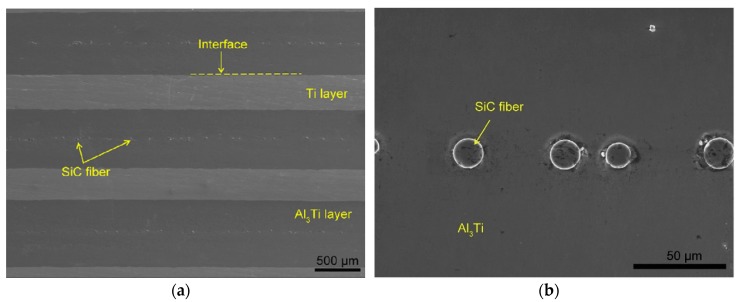
Microstructure images of Ti-(SiC_f_/Al_3_Ti) composite: (**a**) lower magnification and (**b**) higher magnification showing the laminated structure comprised of alternating SiC_f_/Al_3_Ti composite layers and Ti layers.

Recently, the SiC ceramic fiber was introduced into the brittle monolithic intermetallic to improve the mechanical properties of Ti-Al intermetallic alloys [16,17,18,19]. A novel Ti-(SiC_f_/Al_3_Ti) ceramic-fiber-reinforced metal-intermetallic-laminated (CFR-MIL) composite was successfully developed for the first time and preliminary investigations associated with microstructures with tensile mechanical properties, which were carried out in our former work [20]. As shown in Figure 1a, the laminated composite consists of alternating Ti metal layers and fiber-reinforced-intermetallic (FRI) layers. The main portion of FRI layers is intermetallic Al_3_Ti, which is a newly formed phase. The FRI layers also include some SiC fibers distributed along the central section of the layers. It can be observed clearly in Figure 1b. The SiC fibers are bonded with the intermetallic Al_3_Ti and distributed in a line. Ti and SiC fibers are the initial phases, which play different roles in improving the mechanical properties of Ti-(SiC_f_/Al_3_Ti) CCFR-MIL composite. Ti layers can “soften” the laminated composite to provide higher ductility, toughness, and resilience while SiC fibers are the reinforcement that can “stiffen” the composite to obtain higher strength and a certain degree of resilience. The newly developed composite is superior in comprehensive mechanical properties in comparison to the laminated composite without fiber reinforcement. However, the research on this composite is still insufficient.

### 1.2. Research Methodology of Protective Performance of Laminated Composite

**Figure 2:**
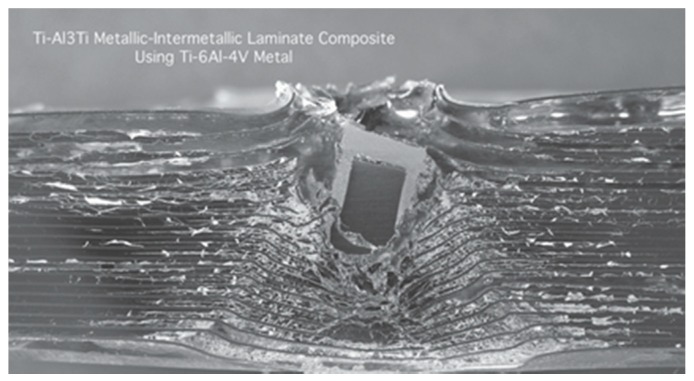
A cross section of the impact location from a ballistic test on an MIL composite.

The investigations on mechanical properties of the laminated composite material during the penetration process may play an important role in predicting the protective performance by which the design of the material can be optimized. There are many problems that occur when using high-velocity armor-piercing resistance tests to study a layered composite. For example, it is difficult to record various experimental phenomena since the penetrating time is short. The experimental results obtained are limited since the material along the loading direction is damaged (shown in Figure 2) [21]. Additionally, the response process and deformation mechanism of the material cannot be described completely.

Compared with the experiment method, the most significant advantage of numerical simulation is to directly display the internal micro-change process of the material, which can provide a basis for the theoretical analysis. In addition, numerical simulations promote the development of the test by reducing costs significantly, which has an important practical significance [22]. According to the discretization method, the numerical simulation method can be divided into a finite difference, a finite element, and element-free methods [23]. Currently, the finite element and the element-free methods play a dominant role in the numerical simulation of composite materials. As is known, the finite element method (FEM) has been extensively applied to solve small-strain issues, which has advantages of high calculation precision and efficiency. However, when it comes to contact problems, the computational efficiency of FEM becomes lower since the existence of the free surface [24]. In particularly when solving large-strain problems such as high-velocity and hypervelocity penetrations, not only the efficiency but also the precision is low. For instance, the finite element will produce mesh distortion when solving problems like sheet metal forming, high-velocity, and hypervelocity penetrations with the Lagrange method. Consequently, the amount of computational work is increased dramatically [25].

Considering the disadvantages of FEM, the research fever of the element-free method has been aroused since the 1990s in the international academic circle of mechanics [26,27]. The element-free method uses a group of points to disperse the solution region and the discrete points are utilized to construct function directly [28]. Therefore, all the operations on mesh such as mesh generation and mesh reconstruction, which have a high computational expense and may reduce the computational precision, are avoided. In this way, not only the computational precision is guaranteed, but also the computational difficulty is reduced when solving large-strain problems. Among many kinds of element-free methods, the smoothed particle hydrodynamics (SPH) method is applied to simulate the large-strain problems such as explosion, high-velocity, and hypervelocity impact. Compared with the other element-free methods, the formulas of the SPH method are simple, which can provide effective treatment of complex boundary problems [29]. However, there is a long-existing disadvantage that exists in solid or liquid simulation. This is known as tensile instability. Additionally, the SPH method has the inherent defect of the element-free method. For example, the computational work is increased due to the complexity of the approximate function in the SPH method. It is more complex to apply essential boundary conditions than FEM since the approximate function of the SPH method doesn’t have a characteristic of interpolation [30].

Considering low computational efficiency and precision when simulating high-velocity impact processes by the SPH method, the mechanical response under low-velocity and quasi-static loading is often applied to investigate the penetration mechanism under a high-velocity impact [31,32,33,34]. The method based on the indentation depth obtained by using a low-velocity impact drop-weight test or simulation to calculate impact energy and residual strength and then to predict anti-penetration performance of the laminated composite material has been widely used in a low-velocity impact study [35]. In this method, a small-strain contact process is utilized to simulate and investigate the large-strain penetration process in which the deep layer material along the loading direction cannot be damaged and the continuity of the material is kept. Therefore, subtle changes of the mechanical situation among the Ti layer, the Al_3_Ti layer, and SiC fibers during the penetration process can be observed more completely and clearly. Therefore, the microstructure strengthening, deformation, and failure mechanisms at the early stage of penetration can be better understood [36] and a large amount of unnecessary computation can be avoided. Especially for the penetration process before the target material is penetrated through, the indentation method can provide a more accurate contact model between the target material and projectile since there is no concern about the residual energy of the projectile [37,38,39].

### 1.3. Previous Works on Elastic-Plastic Mechanical Property and Failure Mechanism of the CCFR-MIL Composite

With regard to the elasto-plastic mechanical properties of the Ti-(SiC_f_/Al_3_Ti) CCFR-MIL composite, a number of researchers have completed research using experiments and numerical simulations. Through a number of experimental studies of elasto-plastic mechanical properties of the CCFR-MIL composite under a low velocity impact [31,40,41,42,43,44,45,46], the parameters that influence the impact response of the CCFR-MIL composite under low velocity impact can be classified into two main groups, which are material-based and geometry-based parameters. Material-based parameters include the type of metals, fibers, and matrix, the layup sequence, the volume ratio of metals and fibers, and the bonding and treatment of interfaces. Correspondingly, the geometry-based parameters include pre-stressing and post-stretching, the size effect of test specimens and impactor, geometry, and velocity. According to the research of many related articles [47,48,49,50], whether a CCFR-MIL composite undergoes aluminum or fiber damage is completely dependent on the layup configuration and aluminum constituents. Additionally, many research studies have been presented about Ti-base CCFR-MIL composite in recent years [51,52,53,54,55]. Nakatani H. et al. reported that, during the low-velocity impact, the damage degree of the laminated composite is mainly predicted according to the deformation of the Ti layer on the non-impacted side [56]. Considering the fatigue performance [54], the CCFR-MIL composite consists of the Ti alloy with high strength but low ductility, which still displays ineffective impact resistance. Fan [57,58] studied the influences of target and impactor size as well as impact location through numerical simulation. The results indicate that the impactor and target size are linearly correlated with a perforation threshold while the impact location has no significant effect. The elasto-plastic mechanical behaviors of the Ti-base CCFR-MIL composite were comprehensively studied in reference [56]. The influence of the interface bonding condition between layers on crack initiation at the non-impacted side is taken into account. In addition, this research also involved the plastic behavior of the metal layer and the damage model for metal, which are also considered in some similar references [57,58,59]. Sadhigi particularly investigated the effect of the element type using the numerical model of the CCFR-MIL composite [60]. Comparing the numerical results with experimental ones, the solid elements display better agreement than shell ones. Tsartsaris [61] simulated a glass-base CCFR-MIL composite to study its low-velocity impact response with LSDYNA 3D-FEM. The numerical method was also applied to model the FML layers including both elastic plastic metal layers and linear-elastic GFRP. Concluding that the dominant shear stress within the material is higher than tensile or compressive stress, metal layers stacked in the middle absorb less energy than the ones placed at two ends. Based on the previous works mentioned above, it is feasible to reveal the microscopic strengthening and deformation mechanisms of the CCFR-MIL composite by investigating the elasto-plastic mechanical properties of such material.

**Figure 3:**
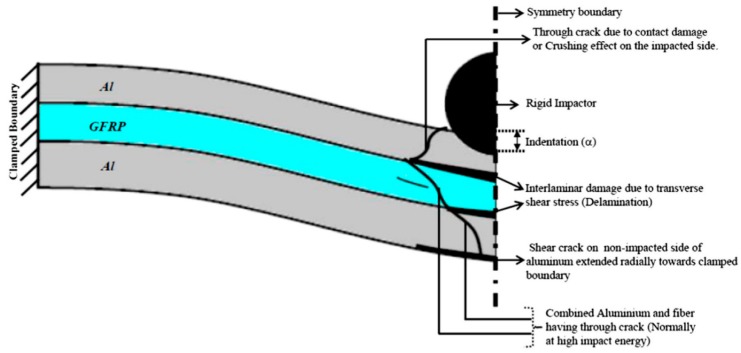
Possible failure modes in clamped CCFR-MIL plate during low-velocity impact test.

It is also worth to point out that many studies have investigated the failure mechanisms of the CCFR-MIL composite during the penetration process. As experimentally analyzed by Richardson Mow et al. [33], impact energy can generate the transverse non-linear dynamic load within the laminated composite material. Additionally, the strength at bonding interfaces between different layers of the CCFR-MIL composite is poor since there is no reinforcement throughout the thickness direction. Therefore, the inter laminar stresses including shear and tension are prone to induce the delamination phenomenon as well as a matrix crack and fiber damage. Focus on the laminated structure of the CCFR-MIL composite, a variety of models have been developed to investigate the mechanical response of impact and failure modes [62,63,64,65,66]. The research developments and findings on this issue were reviewed and illustrated by Chai and Zhu [67] and all the possible modes of failure are summarized in Figure 3 [36]. Comparing the experimental and analytical models for the CCFR-MIL composite material, the 3-D numerical models with an appropriate failure criterion for such laminated composites can align well [68]. With the help of FEM, Linde [69] proposed a modeling approach for the CCFR-MIL composite in ABAQUS and demonstrated the necessity of VUMAT to investigate the failure mechanism. Song [59] simulated the mechanical response of impact for CARAL, which is a typical kind of CCFR-MIL composites, under different levels of impact energy. In this simulation, a 2-D FEM model consists of solid elements for the metal layer and shell elements for the CFRP layer, which was applied. The simulation is based on the Hashin damage criterion and the result of the mechanical response during impact shows a significant agreement with the experimental one. Iannucci L. [70] summarized previous research and classified the modeling methods of failure behaviors in laminated composites into four groups. These groups include (1) the Failure criterion method based on equivalent stress and strain, (2) the Fracture mechanic method based on the energy release rate, (3) the Plasticity or yield surface method, and (4) the Fracture mechanic method based on the degradation degree of material properties. These research achievements on the failure mechanism of CCFR-MIL composite provide a good theoretical foundation to investigate the failure behaviors of such composite during the penetration process. In particular, they have demonstrated that it is a feasible approach for investigating the crack initiation and extension, according to the contact elasto-plastic mechanical behaviors of the composite material at the early stage of penetration.

### 1.4. Development and Advantages of Semi-Analytical Model with Numerical Equivalent Inclusion Method

In the field of small-strain elasto-plastic contact mechanics under quasi-static load, a variety of microstructure-sensitive models have been proposed to analyze material heterogeneity of the hybrid composite laminates through the finite element method (FEM) and the semi-analytical method (SAM). In the case of the Ti-(SiC_f_/Al_3_Ti) CCFR-MIL composite, the intermetallic Al_3_Ti, which is the major portion of the material, can be defined as the matrix. Correspondingly, the SiC fiber and Ti layer can both be considered as inhomogeneities, which are defined as the subdomain of a material with different mechanical properties from those of the matrix [71]. The FEM is flexible and versatile enough to deal with complicated situations. Nevertheless, to build a three-dimensional (3-D) FEM contact model requires a large solution domain (at least 10 times the contact region in each dimension) and very fine mesh. In addition, the execution process is time-consuming. The SAM, in contrast, does not require a large solution domain since it is based on the analytical core solution. Its convenience for solving contact issues has been demonstrated. SAM is in particular applicable to implement the strategy of the equivalent inclusion method (EIM), which was first presented by Eshelby [72]. This transforms an inhomogeneity issue into an inclusion issue coupled with given eigenstrain. According to Mura [71], inclusion is defined as the eigenstrain-possessing sub-domain whose mechanical properties are the same as those of the matrix. Typical examples of eigenstrains are thermal expansion, plastic strain, and the magneto-mechanical strain. It can be divided into two steps when solving an inhomogeneity issue with EIM. First, we substituted inhomogeneities by equivalent inclusions, which possess appropriately assigned eigenstrains to fulfill the requirement that the stress fields caused by equivalent inclusions equal those caused by inhomogeneities. Second, we calculated the stress distributions induced by the eigenstrains. The combination between EIM and the elastic stress solutions of ellipsoidal inclusions [72] has contributed to a number of research studies and method developments. For instance, a high-efficiency approximation method to solve issues involving distributed ellipsoidal inhomogeneities in a 3D half-space was proposed by Zhou et al. [24]. Furthermore, the proposed model was applied to study the influences of particle reinforcement on the rolling fatigue lives of (TiB + TiC)/Ti-6Al-4V composite materials [73].

Recently, aiming to discretize the inhomogeneities with irregular shapes into small cubic elements, the strategy of EIM has been extended to the numerical equivalent inclusion methodology (NEIM) [74,75,76,77,78,79,80]. The stress fields induced by the strains of element cores can be expressed by the basic Galerkin vectors [81] and the cuboidal solution of the stress field in each element has been solved as a closed-form explicit integral kernel for convolution and correlation operations in order to perform the fast solution using the Fourier transform (FFT) [82,83]. A more efficient method was proposed by Wang et al. [84] for the eigenstress calculation in which parallel computing technology was also applied to improve the computational efficiency. The recent research studies on inclusion-related problems were summarized and reviewed by Zhou et al. [85]. The achievements on coupling multiple stress fields and contact elasto-plasticity are also worth mentioning. For instance, by using the NEIM approach mentioned above, Wang et al. [86] took the material inhomogeneity into account when solving the partial-slip contact model. Lately, the effect of inhomogeneities on the stress-strain fields of an elasto-plastic half-space was analyzed by Amuzuga et al. [80]. In addition, a coupled model considering elasto-plasticity, inhomogeneities, and partial slip in contact of materials was constructed by Dong et al. [87].

### 1.5. Research Focus

The CCFR-MIL composite was developed as a material with superior fatigue resistance, excellent impact characteristics, low density, and adequate corrosion resistance property [88,89], which provide a better protective performance for the CCFR-MIL composite during the bullet penetration process. However, the microstructure mechanisms of these property and characteristic advantages are still not clear. For example, the microscopic strengthening mechanism and failure mechanism during penetration. To investigate these mechanisms, we can optimize the microstructure of the CCFR-MIL composite for property improvements, which can further enhance the protective performance during penetration and can also provide significant guidance or direction for the fabrications of similar composite materials. Based on the previous work on related research mentioned above, we know it is a feasible approach to investigate the failure behaviors, microscopic strengthening, and deformation mechanisms of the CCFR-MIL composite through the contact elasto-plastic mechanical behaviors of such composite material at the early stage of the penetration process. Accordingly, in this paper, a high-efficiency SAM based elasto-plastic sphere-plane contact model with NEIM is introduced to numerically simulate and analyze the elasto-plastic behaviors of the Ti-(SiC_f_/Al_3_Ti) laminated composite at the early stage of the penetration process and to conduct in-depth parametric analyses to quantify the influences of the SiC fiber, the Ti layer, and the Al_3_Ti layer on protective performance of the laminated composite for microstructure optimization. The specific research is shown below.

First, the microstructure and interface features were characterized by using a scanning electron microscopy (SEM). Second, the mechanical properties including plane-plane and sphere-plane contact compressive strength of the laminate composite with and without fiber reinforcement were measured via quasi-static compression tests. Third, the accuracy and feasibility of the presented SAM contact model for Ti-(SiC_f_/Al_3_Ti) laminated composite material was validated through the results compared with the compressive stress-strain curves of indentation tests under a spherical indenter and meanwhile, the failure behaviors, microscopic strengthening, and deformation mechanisms at the early stage of the penetration process were also studied through the SAM model. Lastly, several in-depth parametric studies were performed for quantifying the influence of microstructure on protective performance by which way the design of CCFR-MIL composite was optimized.

## 2. Experimental Procedure

### 2.1. Materials Preparation

The metal foil metallurgical technique was adopted in the fabrication of the Ti-(SiC_f_/Al_3_Ti) laminated composite in this work. The original component materials before the reaction include titanium alloy (Ti-6Al-4V) foils (thickness of 0.5 mm), aluminum foils (thickness of 0.7 mm), and SiC fibers (diameter of 10–15 μm). Hereafter, Ti refers to Ti-6Al-4V alloy throughout this paper. It should be indicated that the as-received SiC fibers, which were packed into bunches (500/bunch) initially, were heat-treated at 600 °C for 0.5 h and then rinsed with alcohol to remove the colloidal materials on the surface of fibers before being divided into single ones. Before sintering, the foils were ground by the silicon carbide paper to remove surface oxide layers and then cleaned through the water bath in an ultrasonic cleaning machine for 20 min. After that, all the foils were rinsed with alcohol and then dried.

**Figure 4:**
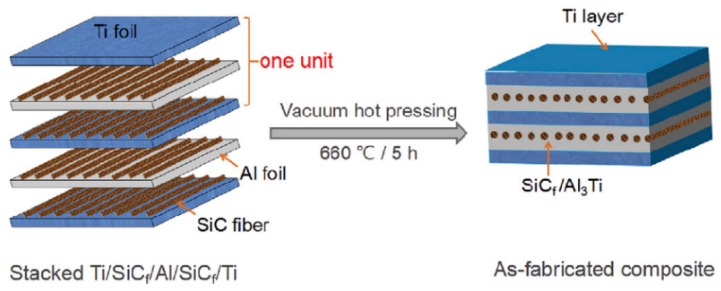
Schematic illustration of the fabrication process of Ti-(SiC_f_/Al_3_Ti) laminated composite.

After these procedures, the foils and fibers were stacked in the order of “Ti-SiC-Al-SiC-Ti,” which was considered one basic “unit,” which was displayed in Figure 4. After all units were stacked together in the right order, the stack was sintered in a vacuum hot pressing furnace (ZRY-10-40, Jin Zhou Huaxin Power Electronica Co., Ltd., Jin Zhou, China) in a high vacuum (~10^−3^ Pa). The temperature was first 620 °C and then increased to 640 °C at a heating rate of 1 °C/min. The temperature was kept for 30 min in these two temperature levels, respectively. In the following step, the temperature was gradually raised to the maximum value of 660 °C in 0.5 h and then held at 660 °C for 5 h. After the sintering process, the laminated composites were cooled down to room temperature along with the furnace. A loading pressure of 3 MPa was imposed on the stack during most of the processing period except for the temperature preservation process at 660 °C. Correspondingly, the Ti-Al_3_Ti laminated composite without fiber reinforcement was also prepared with the same method for comparison.

### 2.2. Microstructural Characterization

**Figure 5:**
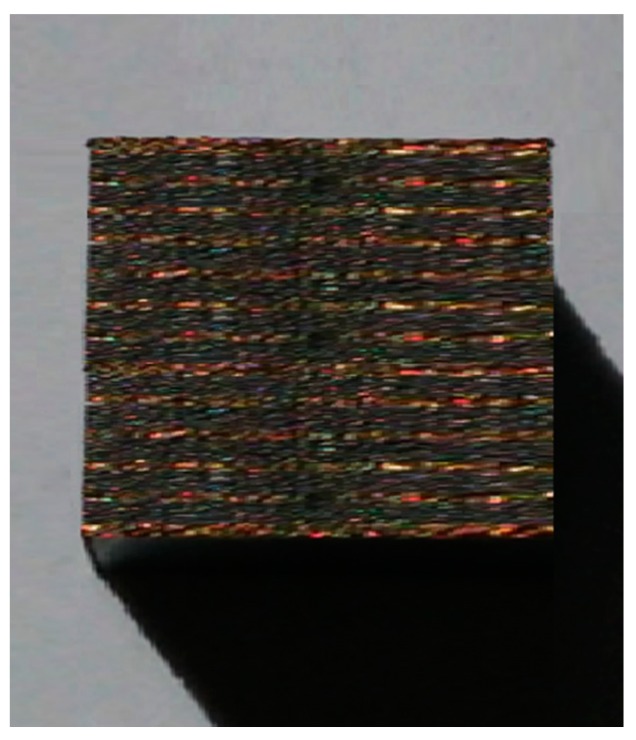
Metallographic specimen configuration of Ti-(SiC_f_/Al_3_Ti) CCFR-MIL composite.

Metallographic specimens were prepared into small cubes with the dimensions of 6 mm × 6 mm × 6 mm, which is shown in Figure 5. They were cut from a CCFR-MIL composite plate by an electrical discharge machine (EDM, Suzhou Baoma, Suzhou, China). The polished surface was perpendicular to the fiber orientation, which could display the laminated structure of the composite. This cross section was prepared first by gradually grinding with 800, 1000, 1500, 2000, 3000 grit silicon carbide papers and then by polishing via the abrasive finishing machine (Wuxi Oubang Machinery Equipment Co., Ltd., Wuxi, China). The volume fraction of each part and microstructure of interfacial reaction zones after hot pressing sintering were observed by using the HITACHI SU-70 scanning electron microscope (SEM, Techcomp (China) Scientific Instrument Co., Ltd., Beijing, China).

### 2.3. Mechanical Testing Methods

**Figure 6:**
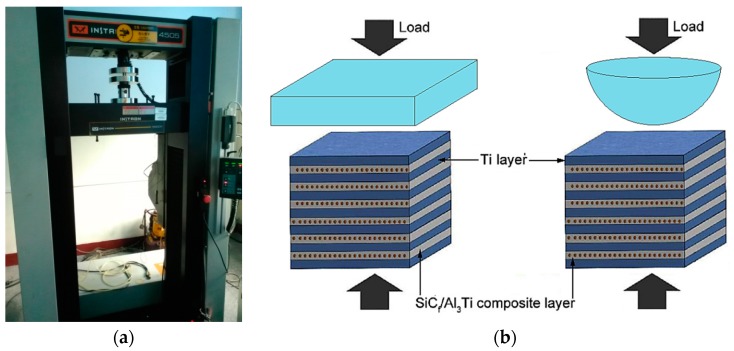
(**a**) Instron 5500R load frame device, (**b**) Specimen configurations employed in compressive tests.

The compression tests were conducted on an Instron 5500R load frame device (shown in Figure 6a, Instron, Shanghai, China) at a strain rate of ~0.001/s at room temperature. The dimensions of the specimens were 6 mm × 6 mm × 6 mm. The loads were applied in a plane-plane and sphere-plane contact mode, respectively, which is shown in Figure 6b. The sphere-plane contact load was applied by a semi-spherical indenter whose diameter was 8 mm. The indenter can be considered as a rigid body. Compared with the plane-plane contact mode, the sphere-plane contact mode can provide more realistic conditions corresponding to the early stage of the penetration process. The compressive strength and failure strain were derived from the measured engineering stress and strain data.

## 3. Elasto-Plastic Contact of Inhomogeneous Materials in a SAM Model with NEIM

### 3.1. Coupling Relationship of Elasticity-Plasticity-Inhomogeneity in Contact Problem

#### 3.1.1. Consistency Condition of Inhomogeneous-Inclusion

Inhomogeneity is a sub-domain of the material whose mechanical properties are different from those of the matrix material. Inhomogeneity may also have an eigenstrain. This is called inhomogeneous-inclusion [71]. For the scenarios in this paper, the eigenstrain in the inhomogeneities is plastic strain and the plastic strain remains constant when solving the inhomogeneity issues. The equivalent inclusion method (EIM) is applied to solve the inhomogeneous-inclusion issue [72]. For the inhomogeneity elements with the number of α,β,γ, the consistency condition is expressed below.

(1) $$C_{ijkl\left[\alpha ,\beta ,\gamma \right]}^{*}\left(\varepsilon_{kl\left[\alpha ,\beta ,\gamma \right]}^{0}+\varepsilon_{kl\left[\alpha ,\beta ,\gamma \right]}-\varepsilon_{kl\left[\alpha ,\beta ,\gamma \right]}^{P}\right)=C_{ijkl}\left(\varepsilon_{kl\left[\alpha ,\beta ,\gamma \right]}^{0}+\varepsilon_{kl\left[\alpha ,\beta ,\gamma \right]}-\varepsilon_{kl\left[\alpha ,\beta ,\gamma \right]}^{P}-\varepsilon_{kl\left[\alpha ,\beta ,\gamma \right]}^{*}\right)$$

where $\varepsilon_{ij}^{0}$ is the elastic strain when the material at the element is homogeneous, $\varepsilon_{ij}$ is the disturbance strain, $\varepsilon_{ij}^{P}$ is the plastic strain, $\varepsilon_{ij}^{*}$ is the equivalent eigenstrain, and $C_{ijkl}$ and $C_{ijkl}^{*}$ are elastic coefficient matrixes of inhomogeneity and matrix, respectively.

According to the reference [71], the plastic strain $\varepsilon_{ij}^{P}$ has a linear correlation with an equivalent eigenstrain $\varepsilon_{ij}^{*}$. Therefore, the disturbance strain $\varepsilon_{ij}$ in Equation (1) can be divided into two parts, which are related to the plastic strain and the equivalent eigenstrain, respectively. This is shown in the equation below.

(2) $$\varepsilon_{ij}=\varepsilon_{ij}^{\left(1\right)}+\varepsilon_{ij}^{\left(2\right)}$$

where $\varepsilon_{ij}^{\left(1\right)}$ and $\varepsilon_{ij}^{\left(2\right)}$ are disturbance strains, which are related to plastic strain and equivalent eigenstrain, respectively. Substitute Equation (2) into Equation (1).

(3) $$\begin{array}{l} C_{ijkl\left[\alpha ,\beta ,\gamma \right]}^{*}\left[\left(\varepsilon_{kl\left[\alpha ,\beta ,\gamma \right]}^{0}+\varepsilon_{kl\left[\alpha ,\beta ,\gamma \right]}^{\left(1\right)}-\varepsilon_{kl\left[\alpha ,\beta ,\gamma \right]}^{P}\right)+\varepsilon_{kl\left[\alpha ,\beta ,\gamma \right]}^{\left(2\right)}\right]\\ =C_{ijkl}\left[\left(\varepsilon_{kl\left[\alpha ,\beta ,\gamma \right]}^{0}+\varepsilon_{kl\left[\alpha ,\beta ,\gamma \right]}^{\left(1\right)}-\varepsilon_{kl\left[\alpha ,\beta ,\gamma \right]}^{P}\right)+\varepsilon_{kl\left[\alpha ,\beta ,\gamma \right]}^{\left(2\right)}-\varepsilon_{kl\left[\alpha ,\beta ,\gamma \right]}^{*}\right] \end{array}$$

Let $\varepsilon_{ij}^{H}=\varepsilon_{ij}^{E}+\varepsilon_{ij}^{\left(1\right)}-\varepsilon_{ij}^{P}$ where superscript *H* means the “homogeneous solution.” Then Equation (3) becomes the formula below.

(4) $$C_{ijkl\left[\alpha ,\beta ,\gamma \right]}^{*}\left(\varepsilon_{kl\left[\alpha ,\beta ,\gamma \right]}^{H}+\varepsilon_{kl\left[\alpha ,\beta ,\gamma \right]}^{\left(2\right)}\right)=C_{ijkl}\left(\varepsilon_{kl\left[\alpha ,\beta ,\gamma \right]}^{H}+\varepsilon_{kl\left[\alpha ,\beta ,\gamma \right]}^{\left(2\right)}-\varepsilon_{kl\left[\alpha ,\beta ,\gamma \right]}^{*}\right)$$

Equation (4) demonstrates how the plastic strain of inhomogeneity is involved with the initial condition. An equivalent eigenstrain is the only unknown. Therefore, Equation (4) can be solved.

#### 3.1.2. Plastic Strain Calculation

The material generates stress and strain under external force. If the stress doesn’t exceed the elastic limit of material, the generated deformation would completely disappear after external force is removed and the material can be restored to its original state. This kind of reversible deformation is defined as elastic deformation. However, if the stress exceeds the elastic limit of the material, the generated deformation cannot be restored to the original state after the external force is removed. This kind of residual deformation is known as irreversible plastic deformation. In this paper, the inhomogeneity and matrix can generate plastic deformation under the external load. Although the material properties of inhomogeneities and matrix are different, the theory and algorithm in this section are applicable to both of them. Accordingly, the parameter symbols in the following paragraphs are not distinguished by the material type.

The von Mises yield criterion is applied to the identify yield. It is considered to occur due to plastic deformation when von Mises stress exceeds yield stress. The total Mises stress in one element is the superposition result of elastic stress, residual stress, and eigenstress, which was caused by inhomogeneities. This is $\sigma_{VM}=\sigma_{ij}^{E}+\sigma_{ij}^{R}+\sigma_{ij}^{*}$. The yield function is expressed by the equation below.

(5) $$f=\sigma_{VM}-\sigma_{Y}=\sqrt{\frac{3}{2}S_{ij}:S_{ij}}-g\left(\lambda \right)$$

where $\sigma_{VM}$ is Mises stress, $\sigma_{Y}$ is yield stress, $S_{ij}$ is deviatoric stress expressed as $S_{ij}=\sigma_{ij}-\sigma_{kk}\delta_{ij}/3$, and g(λ) is the yield stress equation.

For the perfect plasticity material, the yield stress equation g(λ) remains constant and equal to the initial yield stress during plastic deformation. However, in reality, most of the materials possess the characteristic of plastic hardening. In this paper, the isotropic hardening of material is considered and described by the Swift equation, so the relationship between yield stress [90] and plastic strain can be expressed by the equation below.

(6) $$\sigma_{Y}=g\left(\lambda \right)=B{\left(C+\lambda \right)}^{n}$$

where B,C,n are the work hardening parameters related to material property and λ is the equivalent accumulative plastic strain expressed as $\lambda =\sum d\lambda =\sum \sqrt{2d\varepsilon_{ij}^{p}:d\varepsilon_{ij}^{p}/3}$.

The material yields when yield function f(λ)>0. At the same time, the increment of plastic strain Δλ is generated and transforms the yield function back to zero again, which is f(λ+Δλ)=0. A universal integration algorithm, proposed by Fotiu and Nemat-Nasser [91] and improved by Nélias et al. [92], is used to calculate the value of Δλ in each load step. Then the component of increment can be solved by using the plastic-flow rule, which is shown below.

(7) $$\Delta \varepsilon_{ij}^{P}=\left[\lambda^{\left(n+1\right)}-\lambda^{\left(1\right)}\right]\frac{3S_{ij}^{\left(n+1\right)}}{2\sigma_{VM}^{\left(n+1\right)}}$$

Plastic strain is one kind of eigenstrain [75], which means the calculation of residual stress and surface residual deformation generated by plastic strain is the same as the one of eigenstress and surface eigenstrain generated by the eigenstrain, which is unnecessary to restate here.

### 3.2. Solving Method of Elasto-Plastic Contact Model for Inhomogeneous Material

**Figure 7:**
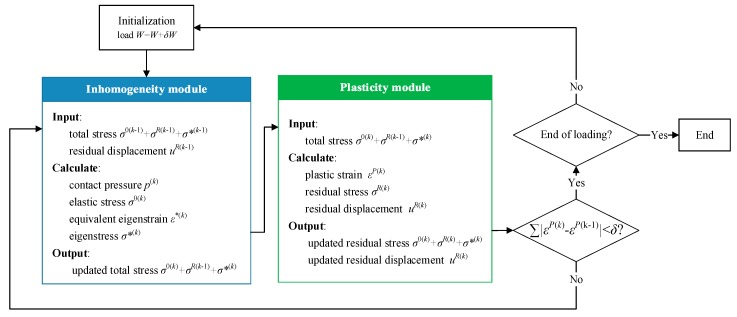
Flow chart of the algorithm and steps for solving inhomogeneous contact elasto-plastic problems.

In the elasto-plastic semi-analytical model of inhomogeneous material, the numerical iteration algorithm is required to solve the equivalent eigenstrain and plastic strain. Then the results of these two parameters are applied to calculate the eigenstress and residual stress. Lastly, the result of the total stress field is obtained. The numerical iteration algorithm to solve the equivalent engenstrain is proposed by Zhang et al. [90,91,92,93]. In general, the total load is divided into several load-steps to increase the concreted force on indenter step by step. The algorithm is divided into two main parts, which include the inhomogeneity module and the plasticity module. The inhomogeneity module is used to solve the equivalent eigenstrain and calculate contact pressure distribution, the elastic stress field, and eigenstress. The input values include the current total stress field (by superposing the elastic stress, residual stress and eigenstress solved in the last iteration step) and surface residual displacement generated by plastic strain. The plastic strain stays constant while the inhomogeneity module is running, which is reflected through the residual stress and surface residual displacement (which are both single-valued functions for plastic strain). They are inputted in the inhomogeneity module and are constant. After the elastic stress and eigenstress in inhomogeneity module are updated, they accompanied with the residual stress uncovered in last iteration step and are all taken into the plasticity module as input values. The main task of the plasticity module is to solve the plastic strain of each element according to Equations (5)–(7). Then the residual stress and surface residual displacement is calculated for the next cycle step. After the plasticity module has finished running, the difference of the equivalent eigenstrain between two adjacent iteration steps is validated whether it satisfies the convergence condition or not. If not, then turn to the inhomogeneity module to begin a new iteration step. If the module reaches the convergence criterion, it then skips to the next load-step. The flow chart of the algorithm is shown in Figure 7.

The inhomogenities would surely induce the disturbance stress when the stress field exists within the material. After the contact load is removed, the elastic stress moves back to zero and the total stress within the material is composed of residual stress and eigenstress. Considering that the current eigenstress is induced by residual stress, the initial stress $\sigma_{ij}^{0}$ should be substituted by residual stress $\sigma_{ij}^{R}$ during the solving process.

### 3.3. Model Validation for Accuracy

In this section, the results of the FEM model with appropriate parameter setup are regarded as a standard and the computational accuracy of the semi-analytical elasto-plastic contact model is validated by comparing results. The model parameter setup such as material properties, load magnitude, and boundary conditions are shown in Table 1 and Table 2, respectively. Three typical cases are employed to compressively validate the presented SAM model, which are: (1) The matrix material is elasto-plastic and inhomogeneity material is hard elastic. To validate whether or not the model can accurately calculate the influence of inhomogeneities-induced disturbance stress on matrix plastic deformation, the matrix material chosen was elastic and the inhomogeneity material was soft elasto-plastic. To validate whether or not the model can accurately calculate the plastic deformation within inhomogeneities, the matrix material chosen was elasto-plastic and inhomogeneity material was soft elasto-plastic. To validate whether or not the model can accurately calculate the coupling relationship between material inhomogeneity and plasticity, the elasto-plastic materials discussed above require exponential type isotropic hardening.

**Figure 8:**
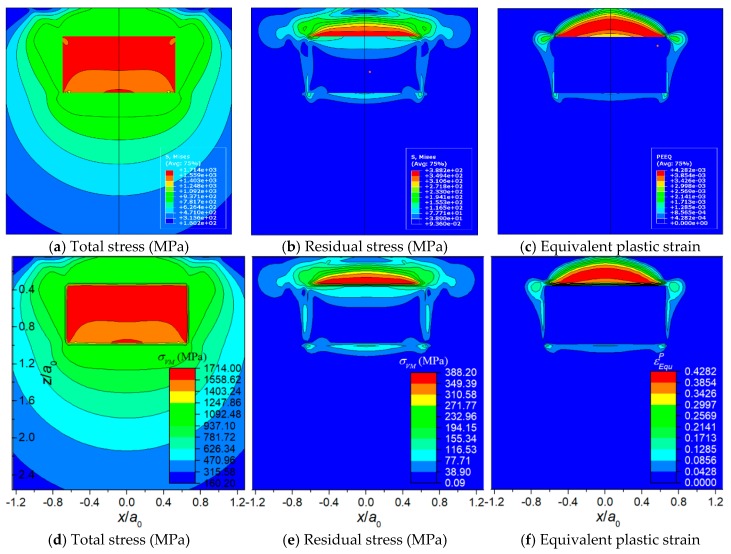
Case 1: The inhomogeneity is stiff and elastic and the matrix material is elasto-plastic. (**a**–**c**) results of FEM, (**d**–**f**) results of SAM.

**Figure 9:**
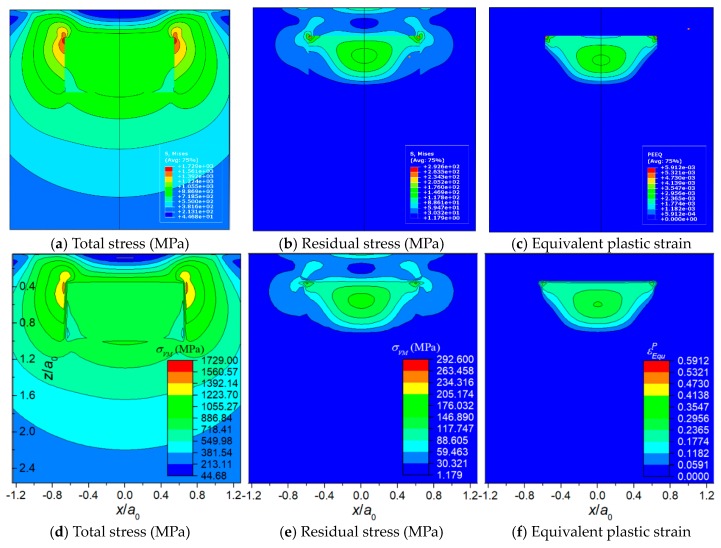
Case 2: The inhomogeneity is compliant and elasto-plastic while the matrix material is elastic. (**a**–**c**) results of FEM and (**d**–**f**) results of SAM.

**Figure 10:**
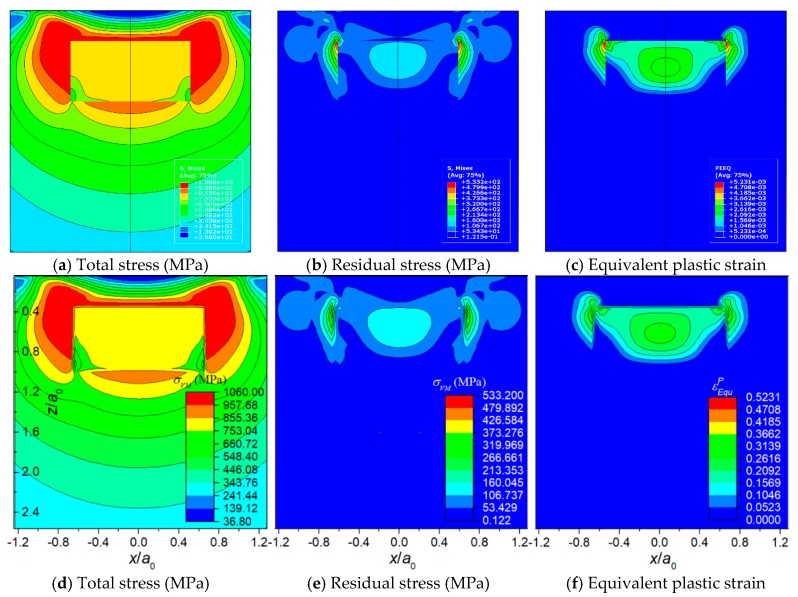
Case 3: The inhomogeneity is compliant and elasto-plastic. Matrix material is elasto-plastic. (**a**–**c**) results of FEM and (**d**–**f**) results of SAM.

**Figure 11:**
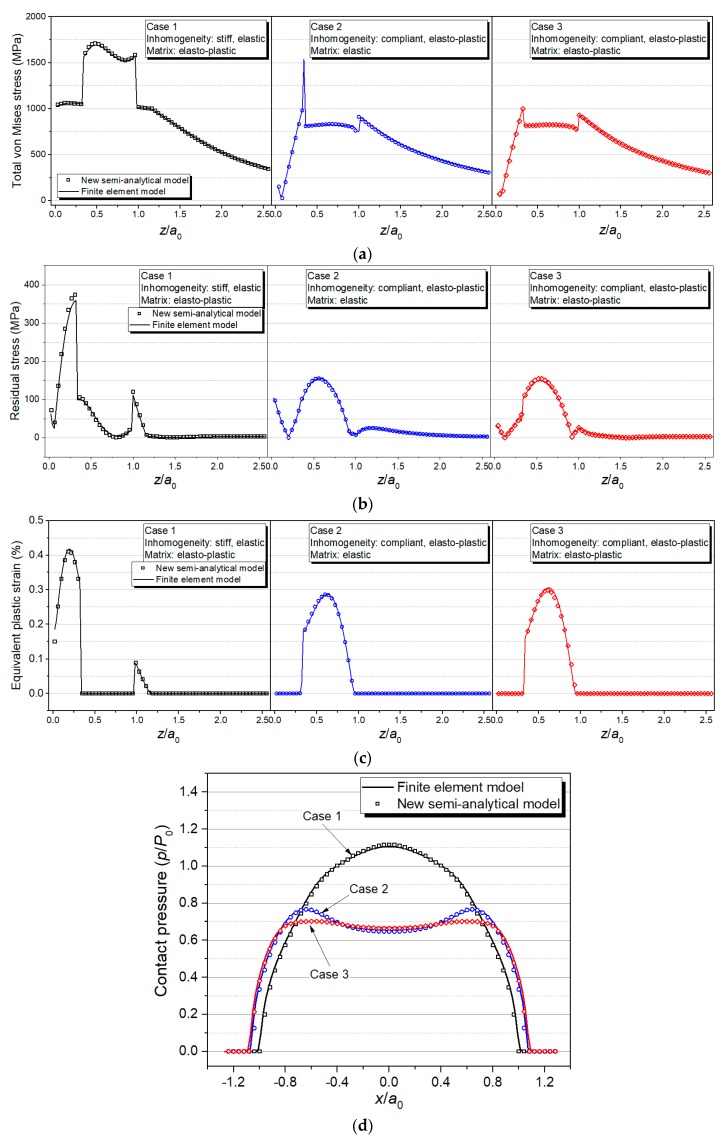
Detailed comparisons of the results of FEM and SAM along the *z*-axis or the *x*-axis: (**a**) total von Mises stress, (**b**) residual von Mises stress, (**c**) equivalent plastic strain, and (**d**) contact pressure.

Figure 8, Figure 9 and Figure 10 displays the results of the three typical cases solved by the FEM model and the SAM model, respectively. The results include total stress, residual stress, equivalent plastic strain, and contact pressure distribution. All the contour maps employ an identical scale to conduct intuitional comparison. Figure 11 shows the detailed comparisons between the results of FEM and SAM along the *z*-axis or the *x*-axis. The results indicate that the semi-analytical elasto-plastic contact model can calculate the results as accurately as the FEM model while the mesh density of it is much lower than the one of the FEM model.

### 3.4. Model Validation for Efficiency

**Figure 12:**
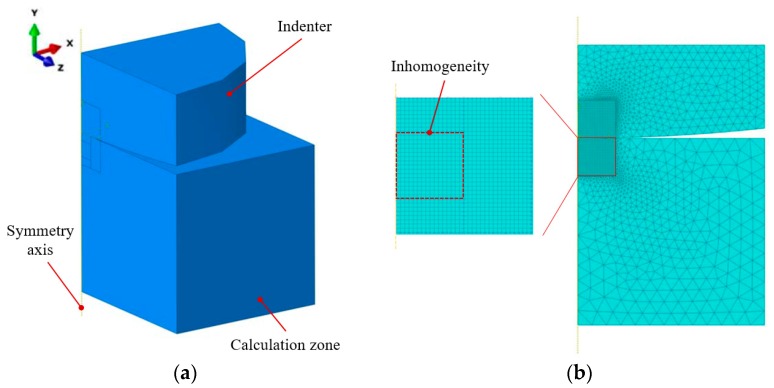
Schematic of (**a**) finite element model and (**b**) mesh local refinement.

Compared with FEM, the SAM described above shows a great efficiency advantage when tackling elasto-plastic contact problems of inhomogeneous materials without losing any accuracy, which is validated in this section. The contact between a spherical indenter and half-space contain single rectangular inhomogeneity simulated by FEM and SAM models, respectively, and execution time of the two models are compared. The upper surface of the rectangular inhomogeneity is parallel to contact surface and the central point of inhomogeneity is right under the contact point, which indicates the geometric model for the three cases possessing a geometrical feature of axial symmetry. Therefore, the cases can be simulated by a 3-D quarter FEM model, which is shown in Figure 12a. However, in most of the practical issues such as the composite materials containing randomly distributed inhomogeneities, a complete 3-D FEM model is required. The typical cases solved in this section is the same with the ones in Section 3.3. Therefore, the material properties and model parameters are also displayed in Table 1 and Table 2. The size of elements in the local refinement area of the FEM model is set as 4 μm, which is shown in Figure 12b. This is identical to the element size of the SAM model. Since building a 3-D FEM contact model requires a large solution domain (far larger than contact region) to guarantee the computational precision, which means even though only a quarter of the solution domain is modeled in FEM, the total number of computational grids is still up to 253,517, which is approximately the same with the grid number ($64^{3}=262,144$) in the SAM model. All the cases in this section are solved with Intel^®^ Core^TM^ i7-8700 CPU.

**Figure 13:**
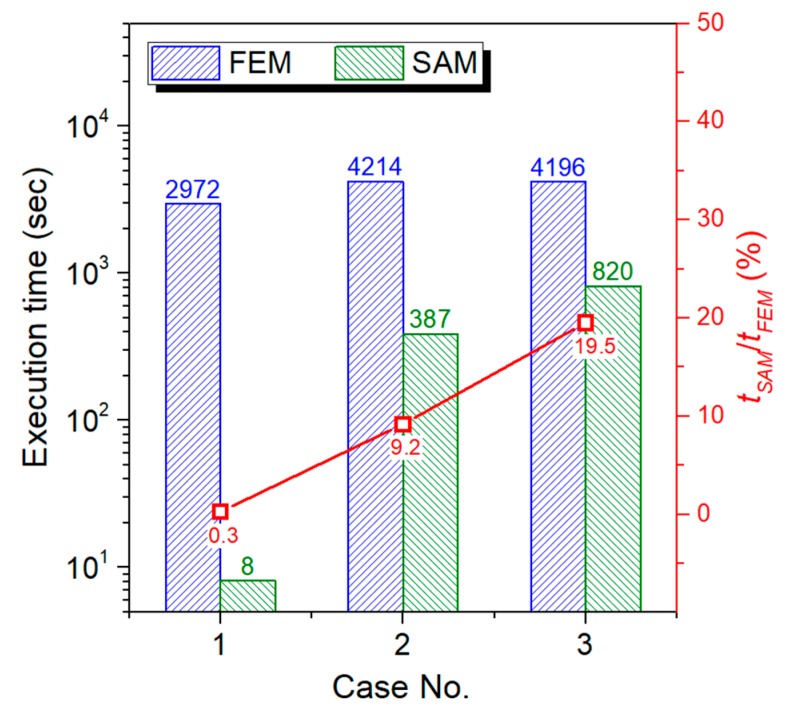
Comparison of execution time of FEM and SAM.

The execution time of FEM and SAM models are shown in Figure 13, which implies that the computation speed of SAM model is much higher than the one of the FEM model when grid numbers of the two models are drawn close to each other and the max ratio between the two execution times (expressed as *t_SAM_*/*t_FEM_*) is only 19.5%.

According to the results in Section 3.3 and Section 3.4, the advantages of the semi-analytical elasto-plastic contact model with a numerical equivalent inclusion method are validated, which are: (1) SAM model can provide the same precision with the FEM model at relatively sparser grids. (2) The efficiency of the SAM model is higher when the grid numbers of two models are almost the same.

## 4. Results Comparison between SAM Model and Indentation Test for Ti-(SiC_f_/Al_3_Ti) CCFR-MIL Composite

### 4.1. SAM Model for Ti-(SiC_f_/Al_3_Ti) CCFR-MIL Composite

Aiming to further validate that the presented SAM model is suitable to simulate the Ti-(SiC_f_/Al_3_Ti) CCFR-MIL composite, the SAM model in a reasonably dense mesh was validated by simulating the contact between a rigid spherical indenter and a half-space Ti-(SiC_f_/Al_3_Ti) CCFR-MIL composite material containing layered inhomogeneity of Ti and cylindrical inhomogeneities of the SiC fiber. The results are compared and discussed with the ones of the compressive indentation test.

**Figure 14:**
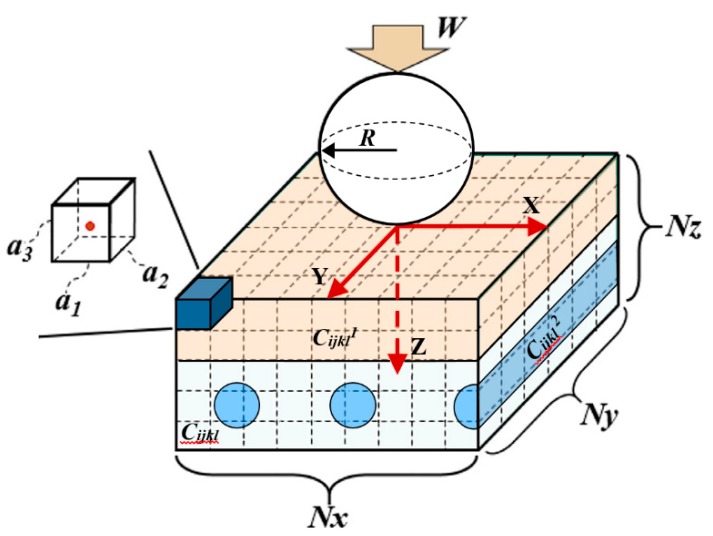
Inhomogeneous material subjected to sphere-plane contact loading containing a layered inhomogeneity and multiple cylindrical inhomogeneities. The element at the top left corner of the half space is magnified to display the definition of an element by its edges, *a*_1_–*a*_3_.

**Figure 15:**
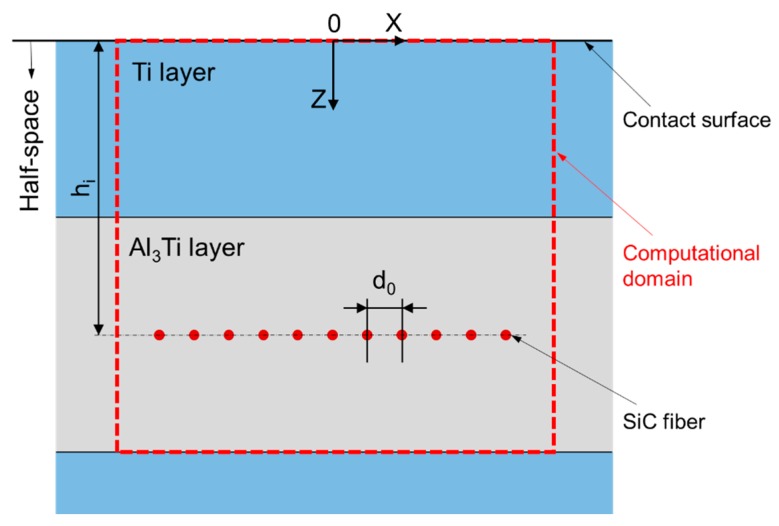
XOZ cross section of 3-D SAM model for validation.

In the SAM, as shown in Figure 14, the computational domain is bounded by a Cartesian coordinate system where the *x*-axis and the *y*-axis are parallel to the surface of the half space and the *z*-axis points to depth direction. The origin, *O*, of the coordinate system is superposed with the contact point. The entire calculation zone including both the inhomogeneities and matrix materials is discretized into *Nx* × *Ny* × *Nz* cubic elements. A concentrated loading force, *W*, is applied on a spherical indenter whose radius is *R*. Material properties of each element are assigned according to the core location whether in inhomogeneities or the matrix. The elastic coefficients of matrix, layered and columnar inhomogeneities are $C_{ijkl}$, $C_{ijkl}^{1}$, and $C_{ijkl}^{2}$, respectively. The major parameters are listed in Table 3. The geometric parameters were determined according to the microstructure images of the Ti-(SiC_f_/Al_3_Ti) CCFR-MIL composite in Electron microscope scanning experiment. The life-size cross section view of the SAM model is shown in Figure 15. The center axis of the indenter coincides with the *z*-axis. In addition, based on the results of the compressive strength test in Section 5.2, the safety loading magnitude ranged from 0 kN to 21.6 kN. Failure behaviors first appear in the Al_3_Ti layer during the compression test. This is due to the high ductility and toughness of Ti and the high strength of SiC. These two materials do not fail easily under a certain load. Therefore, in the current model, the Al_3_Ti layer is elastio-plastic while SiC fibers and Ti layer are assumed to be elastic for simplicity. In addition, the plasticity in the Al_3_Ti matrix layer is of the greatest concern given that the matrix layer is the major portion of the material system and its properties can approximately represent the whole material.

### 4.2. Indentation Test for Ti-(SiC_f_/Al_3_Ti) CCFR-MIL Composite

The compressive indentation tests are conducted on the same equipment and conditions as the compressive test with spherical indenter in Section 2.3. The maximum load within the safety loading magnitude needs to be limited, which is under 21.6 kN, according to the compressive stress-strain curves in Section 5.2. The tests are performed three times on each kind of specimens (Ti-(SiC_f_/Al_3_Ti) CCFR-MIL and Ti-Al_3_Ti MIL specimens) to avoid the statistical error.

The results of the compressive indentation test were displayed in the form of load-strain curves. When the indenter touched the specimen and began to apply a load, the rigid-body motion between the two solids until the loading finished is *S_l_*. It is known that the height of specimens *Hs* was 6 mm. Therefore, the total strain of indentation when the load was on, $\varepsilon_{t}^{c}$ is also the maximum total strain of contact surface expressed as $\varepsilon_{t}^{c}=S_{l}/H_{s}$. Then, when loading terminated and began to unload, the rigid-body motion between the two solids until the unloading finished is *S_ul_*. Therefore, the maximum plastic strain of residual indentation $\varepsilon_{p}^{c}$ is $\varepsilon_{p}^{c}=(S_{l}-S_{ul})/H_{s}$. As shown in Figure 15, the computational domain of the current model only has one “Ti-Al_3_Ti-SiC-Al_3_Ti” unit. Correspondingly, for scaling up the results of the compressive indentation test to compare with the results from the current model more clearly, the test specimens whose dimensions were 6 mm × 6 mm × 6 mm comprised more or less 12 “Ti-Al_3_Ti-SiC-Al_3_Ti” units are layered along the loading orientation. All key characteristics have been taken into account for the validation issue. For instance, with and without SiC fiber inhomogeneities, stiff (*E_SiC_* > *E_Al__3Ti_*) and compliant (*E_Ti_* < *E_Al__3Ti_*) inhomogeneities, and the deepest layer of the Al_3_Ti matrix material can also deform plastically under the maximum load. Therefore, the solutions can comprehensively test the proposed model for the Ti-(SiC_f_/Al_3_Ti) composite.

### 4.3. Results Comparison and Discussion

**Figure 16:**
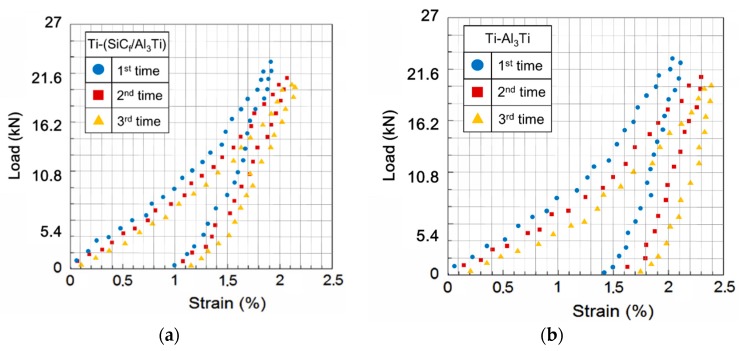
Original data of compressive load-strain curves solved with indentation test under spherical indenter: (**a**) Ti-(SiC_f_/Al_3_Ti) CCFR-MIL specimens and (**b**) Ti-Al_3_Ti MIL specimens.

The original data of compressive load-strain curves solved with the indentation test under a spherical indenter for the Ti-(SiC_f_/Al_3_Ti) CCFR-MIL and Ti-Al_3_Ti MIL specimens are shown in Figure 16a,b, respectively. It can be seen that there are some unreasonable values, which show large discreteness due to experimental uncertainties. Given that, the final result is obtained by removing the unreasonable values and then average the three sets of original data by considering statistical error. The final result is displayed in Figure 16.

**Figure 17:**
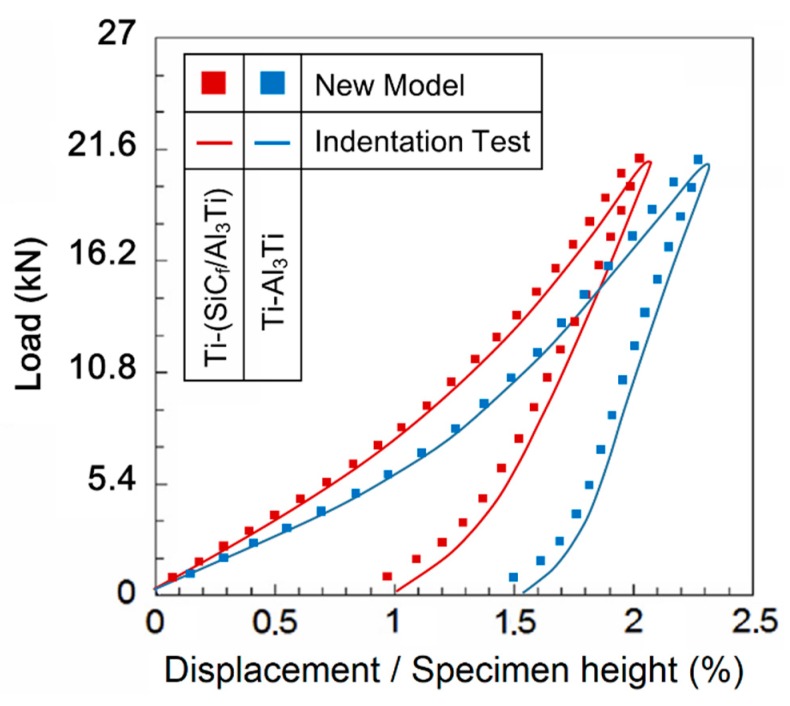
Results of the compressive load-strain curves solved with the 3D SAM model and indentation test under a spherical indenter.

Figure 17 plot the load-strain curves solved with the proposed SAM model and the compressive indentation test. The whole variant trends of the results from the two methods are identical except for some system deviations in which the total strains in the new model is slightly lower than in the corresponding values in the indentation test such as in the maximum plastic strain of residual indentation $\varepsilon_{p}^{c}$ when the load was off. This is due to the different forms of the loading region in the two methods. For the numerical model, the load was applied on a half-space region, which is closer to the actual situation considering the size ratio between the bullet and armor plate. However, the specimens loaded in the compressive indentation test were small cubes, which have four free surfaces around. Therefore, the specimens are “softer” than the ideal half-space material since the deformation is easier to extend out through the free surfaces around. Moreover, owing the existence of friction in the test machine, the elastic deformation could not fully recover when compared to the numerical model when the load was removed, which contributed to the higher deviations on the maximum plastic strain of residual indentation. The maximum deviation is less than 8% and a good agreement was achieved. Therefore, the presented SAM model is validated to be a feasible approach to simulate the Ti-(SiC_f_/Al_3_Ti) CCFR-MIL composite under a certain compressive load.

Compared with the load-strain curve of Ti-Al_3_Ti, the maximum total strain of Ti-(SiC_f_/Al_3_Ti) when the load was on is 10% lower and the plastic strain of the residual indentation is 35% lower. Suggesting that SiC fibers play an important role in improving the strength and resilience of the laminated composite, the microscopic mechanism is studied in Section 5.3.

## 5. Results and Discussion

### 5.1. Microstructure Observation and Characterization

**Figure 18:**
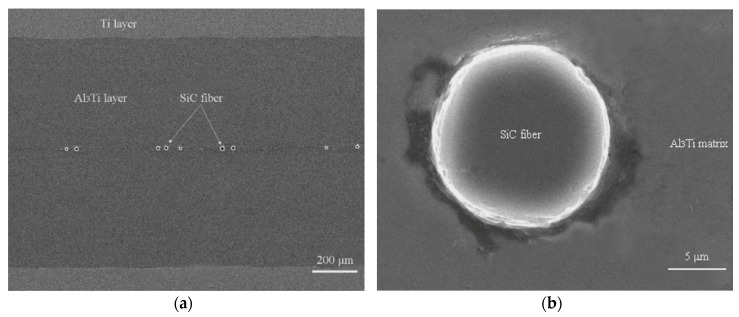
SEM image for the laminated structure of Ti-(SiC_f_/Al_3_Ti) composite: (**a**) lower magnification and (**b**) higher magnification.

Figure 18a displays the typical layered structure of the CCFR-MIL composite. It can be observed that the CCFR-MIL composite consists of the Ti, intermetallic Al_3_Ti layers, and SiC fibers distributed along the central section of the Al_3_Ti layers. Ti and SiC fibers are initial phases, which are the original structural elements before reacting. The intermetallic Al_3_Ti is the newly formed phase that is more thermodynamically and kinetically favored than the other formations of aluminides during the direct reaction of Al with Ti, which is similar to that in the Ti/Al_3_Ti laminate composite [13]. The formation mechanisms of Al_3_Ti through the reaction of Al and Ti have been investigated systematically by D.J. Harach and K.S. Vecchio [9]. Intermetallic Al_3_Ti formation involved interfacial diffusion behavior between solid Ti and liquid Al. The reasons to form the only Al_3_Ti in Ti/Al_3_Ti MIL composites rather than other aluminides such as Ti_3_Al and TiAl can be found elsewhere [94,95].

The volume fraction of residual Ti (*f_Ti_*) was calculated by the equation below.

(8) $$f_{Ti}=\frac{n_{Ti}\cdot h_{Ti}}{n_{Ti}\cdot h_{Ti}+n_{Al_{3}Ti}\cdot h_{Al_{3}T}}$$

where the *n_Ti_*, *n_Al__3Ti_,* and *h_Ti_*, *h_Al__3Ti_* are the number and thickness of Ti and Al_3_Ti layers, respectively. The volume fraction of Ti layer in CCFR-MIL composite solved with Equation (8) is ~22%. The volume fraction of the SiC fibers is difficult to calculate accurately, but it can be estimated approximately according to the diameter and number of SiC fibers, which is around ~1%.

Figure 18b displays the interfacial characteristics between the SiC fiber and the Al_3_Ti intermetallic alloy. Note that the circular interface surrounding the SiC fiber is not complete and there are interstices and gaps in some parts of the interface. This observation indicates that bonding types between the fiber and Al_3_Ti include both the metallurgical reaction and the mechanical joint.

### 5.2. Mechanical Properties of the Ti-(SiC_f_/Al_3_Ti) Composite

**Figure 19:**
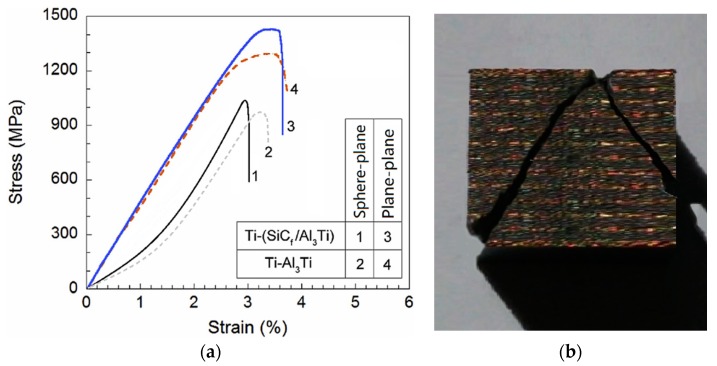
(**a**) Compressive stress-strain curves for Ti-(SiC_f_/Al_3_Ti) and Ti-Al_3_Ti laminated composites at different contact mode of loading and (**b**) the damaged specimen after material failure under sphere-plane contact load.

The results of mechanical tests are summarized in Table 4. It is noted that the compressive strengths (under plane-plane and sphere-plane contact mode of loading, respectively) of Ti-(SiC_f_/Al_3_Ti) CCFR-MIL composite have all improved due to the introduction of SiC fibers, which is compared to the Ti-Al_3_Ti MIL composite without fiber reinforcement. It is apparent that, when the plane-plane contact mode of loading is applied, the compressive strength of the CCFR-MIL composite is ~1453 MPa, which is 10% higher than that of the MIL composite. This result is extracted from the compressive stress-strain curves (3 and 4) plotted in Figure 19a. In addition, the failure strains of CCFR-MIL and MIL composites are approximately equal. Together, these two results suggest the strength of the fiber reinforced laminated composite is remarkably enhanced and the ductility is well maintained, which provides a feasible approach for solving the contradictory issue of strength and ductility. Similar studies were also formerly presented by Han et al. [96] and Wang et al. [97].

Under the load in the sphere-plane contact mode, the compressive strength of the MIL composite (~987 MPa) is only slightly lower than that of the CCFR-MIL composite (~1051 MPa). This indicates that the reinforcement effect of SiC fibers on strength are almost the same under the sphere-plane and the plane-plane contact mode loading. It’s worth pointing out that, due to the fact that the contact surface is not flat, the compressive strength mentioned here is an average value, which stands for the average stress of the contact surface induced by load. The introduction of the concept of average compressive strength is meant to better understand the mechanical property of the materials under the sphere-plane contact mode. This does not have the same meaning as the compressive strength mentioned under a plane-plane contact mode. Moreover, the damaged specimen after material failure under the sphere-plane contact load is shown in Figure 19b. It can be observed that crack initials from the center of the contact surface owes to the loading of the spherical indenter and then extends to the side boundaries of the specimen, which causes the final failure of the material. This suggests that the failure behaviors of the CCFR-MIL composite are not only related to the microstructure but also to the loading contact mode. It is noted that under the sphere-plane mode, the compressive strengths of the two composites with and without fiber reinforcement are respectively lower than those under plane-plane mode. This is because the sphere indenter would apply a concentrated force and induce higher plastic strain concentration in the loaded material, which leads to earlier crack initiation and earlier material failure. In addition, noting that the failure strain of the CCFR-MIL composite is 6% lower than that of the MIL composite under a sphere-plane contact mode, the two values are almost the same under a plane-plane contact mode. This shows that the SiC reinforcement effect for strength is still apparent while the effect for ductility is not as clear as that under plane-plane contact load. It can be inferred that the reinforcement effect of SiC fibers on the ductility of the composite is significant when under a uniform normal contact load. While under a sphere-plane contact load, the material around the indentation is subject to a tangential force from the spherical indenter. This force pushing the material away horizontally expends the crack at the contact point and leads to the final material failure. The SiC fibers can only prevent this kind of failure behavior in one horizontal direction since they are continuous in one horizontal direction and discretely distributed in another. Accordingly, the SiC reinforcement effect on mechanical properties of the material under the sphere-plane loading contact mode is more complicated, which is further studied in the following sections.

### 5.3. Basic Contact Plasticity Behaviors of Ti-(SiC_f_/Al_3_Ti) Composite under Varying Loads

The plastic region evolutions of the SAM model for the Ti-(SiC_f_/Al_3_Ti) composite in Section 4.1 that contains inhomogeneities of SiC fibers and Ti layer are analyzed in this section to reveal the failure behaviors, microscopic strengthening, and deformation mechanisms of the material at the early stage of penetration by using the equivalent plastic strain in the Al_3_Ti matrix at the vicinity of the SiC fibers and Ti-Al_3_Ti interface. The equivalent plastic strain, which can represent the magnitude of plastic deformation concentration within the material, is expressed in Equation (9). The comparison between plastic strain fields with and without SiC inhomogeneities also reveals the reinforcement effects of SiC fibers.

(9) $$\varepsilon_{Eq}^{P}=\sqrt{\frac{2}{3}[{\left(\varepsilon_{11}^{P}-\varepsilon_{22}^{P}\right)}^{2}+{\left(\varepsilon_{22}^{P}-\varepsilon_{33}^{P}\right)}^{2}+{\left(\varepsilon_{33}^{P}-\varepsilon_{11}^{P}\right)}^{2}+2({\left(\varepsilon_{11}^{P}\right)}^{2}+{\left(\varepsilon_{23}^{P}\right)}^{2}+{\left(\varepsilon_{13}^{P}\right)}^{2})]}$$

**Figure 20:**
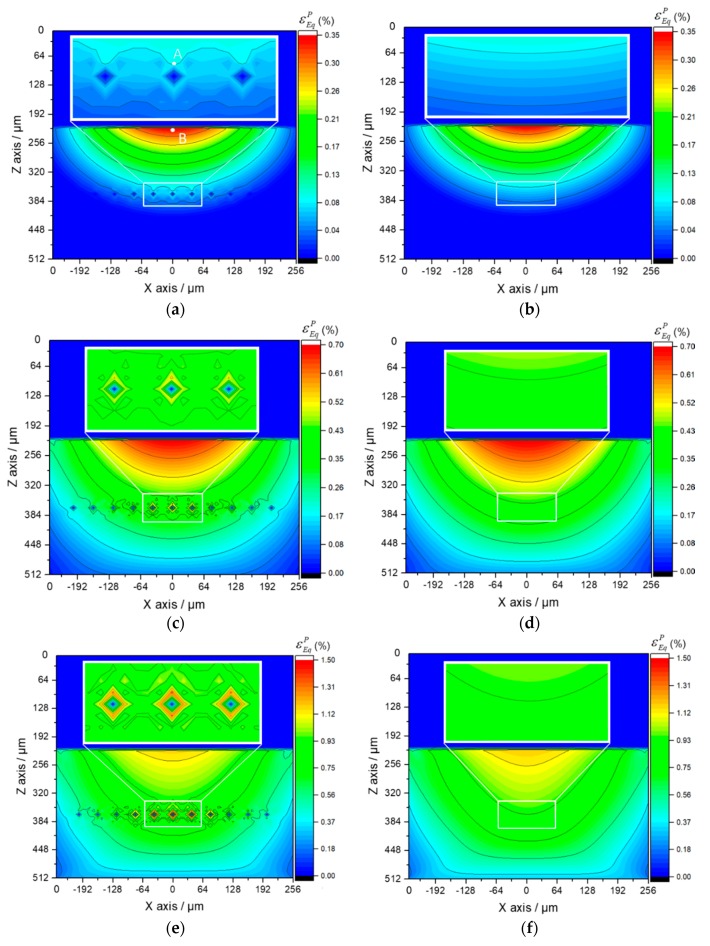

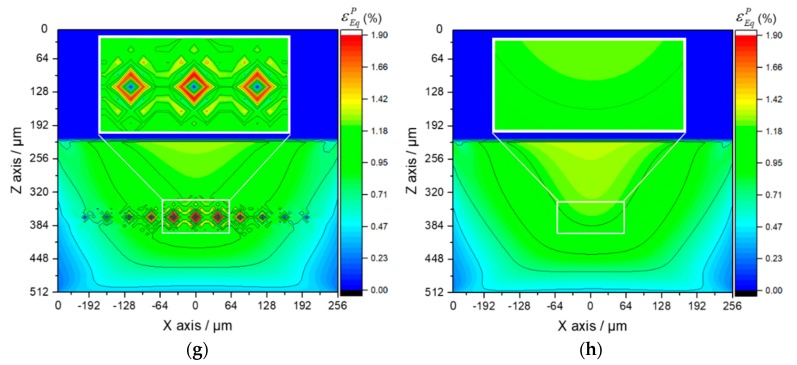
Plastic region evolution in the cross section XOZ of Ti-(SiC_f_/Al_3_Ti) (**a**,**c**,**e**,**g**) and Ti-Al_3_Ti (**b**,**d**,**f**,**h**) laminated composites, with varying load: (**a**,**b**) 5.4 kN, (**c**,**d**) 10.8 kN, (**e**,**f**) 16.2 kN, (**g**,**h**) 21.6 kN.

**Figure 21:**
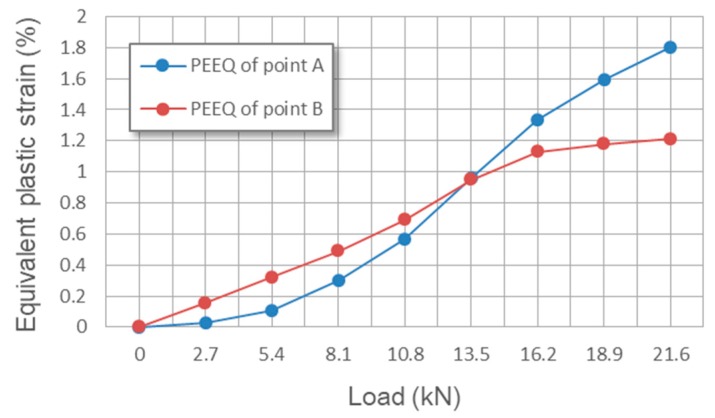
Variations of the equivalent plastic strains at points A and B of Ti-(SiC_f_/Al_3_Ti) with different loads.

Considering that the value of total strain includes a large proportion of elastic strain, the plastic strain is contoured to display the difference of the strain status among each part in the Al_3_Ti matrix material more clearly. It is known that the maximum plastic strain concentration (MPSC) area is also where cracks may initiate and extend from, which leads to the final material failure. According to the plastic region evolution in cross section XOZ of Ti-(SiC_f_/Al_3_Ti), which is shown in Figure 20a,c,e,g, the MPSC in the Al_3_Ti layer first occurs in the center of the interface between the Ti and Al_3_Ti layer (point B). As the load increases, it is substituted by the closest location to the upper boundary of the central SiC fiber (point A) and then extends along depth around the fiber as the load continues increasing. By indicating the location where the cracks may initially form, extend from, and lead to the final material failure under continuous increasing load, also reveals the failure mechanism at the early stage of bullet penetration process. The variations of the equivalent plastic strains at point A and B with different loads are plotted in Figure 21. Apparently, the equivalent plastic strain (PEEQ) of point A increases slowly when compared to that of point B at the beginning of loading while, after the plastic strain concentration area moves down to the SiC fiber string with the increasing load, the PEEQ of point A grows faster than that of point B and exceeds this point at the load of 13.3 kN. This occurs because, at the beginning of the contact loading process, most of the concentrated load is absorbed by the soft Ti layer to generate deformation within this layer. Therefore, the plastic strain only concentrates on the shallow layer of the Al_3_Ti layer around point B and cannot extend to the deeper layer around point A. Accordingly, the PEEQ of point A grows slower than that of point B during the early stage of loading. With the increasing amount of load, the Ti layer deforms to the max but will not fracture due to the high ductility. Therefore, a more concentrated load is imposed on the Al_3_Ti layer. Since the Al_3_Ti is much harder than Ti, the concentrated loading force propagates sooner to the deeper layer of the Al_3_Ti around point A, which means the PEEQ of point A grows faster and faster. It should be noted that the matrix material Al_3_Ti at point A is subject to not only the normal stress from the load above but also to the tangential stress induced by SiC fibers as foreign bodies block the material from moving deeper. The continuous fibers are subject to a non-uniform force under a sphere-plane contact mode of loading, the middle part of the fiber bear the load while the two ends do not. Therefore, the resilience of the high-strength SiC fiber would create considerable tangential stress at the middle part of fiber to separate the material at point A. Since the diameter of fiber is much smaller than that of the spherical indenter, the tangential stress at point A is much higher than that at point B. Therefore, the PEEQ of point A is higher than that of point B under the same normal load as long as the concentrated loading force would propagate to the deeper layer around point A, which explains the phenomenon in Figure 18 beyond 13.5 kN. On the whole, the PEEQ of point A is more susceptible to the change of concentrated load than that of point B. In comparison, for the Ti-Al_3_Ti composite, the MPSC in the Al_3_Ti layer is always in the center of the interface between the Ti and Al_3_Ti layer, which is shown in Figure 20b,d,f,h. This suggests that the failure mechanism of the Ti-Al_3_Ti MIL composite is changed due to the introduction of the SiC fiber reinforcement.

By comparing the images of Figure 20a,b, it was noted that, at the beginning of loading, the plastic strain distributions of the materials with and without the reinforced SiC fiber are almost the same. Then, as the load increases, which is shown in Figure 20c, the plastic strain concentration area moves deeper along the loading direction and is getting closer to the SiC fiber string. Therefore, it can be observed that the high strength SiC fibers, which have mostly an elastic strain, can “pull” the strain contours and keep them from propagating to the deeper layer. By comparing Figure 20a,b,g,h, respectively, the “pulling effect” is more clear when the load goes up. This indicated that the SiC fibers do make a difference in improving the strength of the Ti-Al_3_Ti MIL composite by effectively preventing the plastic strain from extending to the deeper layer. In addition, this improving effect is more obvious at large load than at small ones. Correspondingly, when the load is removed, the SiC fibers with elastic deformation can provide more of a restoring force than the matrix material of Al_3_Ti. This explains the phenomenon in Figure 17, which showed how the SiC fibers improve both strength and resilience of the laminated composite.

The comparison between the equivalent plastic strain fields with and without SiC fiber reinforcement indicated that the SiC fiber string is also a local plastic strain concentration raiser even though it can lower the global strain. This explains the phenomenon in Figure 19a and the reason why the SiC fiber string can lower the failure strain while alsostrengthening the material. When the fracture or breakage occur on SiC fibers, the high plastic strain concentration around the fibers would extend to other parts of the Al_3_Ti layer immediately. This is the reason why the failure process of Ti-(SiC_f_/Al_3_Ti) is shorter than that of the Ti-Al_3_Ti layer, which is shown in Figure 19a. The strengthening effect of the SiC fiber string is connected with the geometric parameters of SiC fibers and the buffer effect of the Ti layer on the load is related to the volume fraction of Ti. Accordingly, in-depth parametric studies are necessary. These are carried out in the following section.

### 5.4. Parametric Studies

#### 5.4.1. Effect of SiC Fibers’ Distribution

It is well known that the appropriate distribution of SiC fibers plays an important role in improving the mechanical properties of the fiber reinforced composites. Therefore, based on the SAM model for the Ti-(SiC_f_/Al_3_Ti) composite in Section 4.1, the plastic strain fields in a half-space Ti-(SiC_f_/Al_3_Ti) composite with various center distances between adjacent SiC fibers are analyzed in this section in order to find out the optimal value for the best mechanical properties. The magnitude of the load is fixed at 21.6 kN.

**Figure 22:**
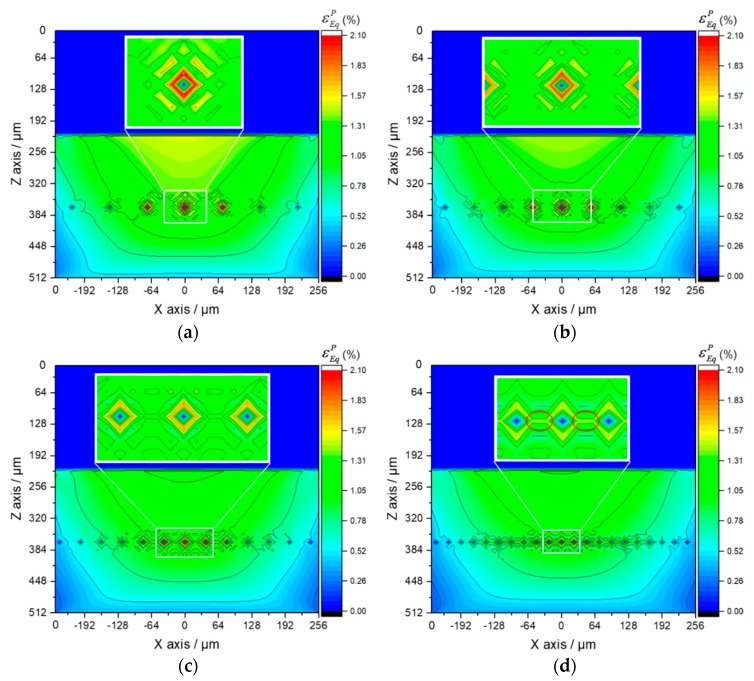
Equivalent plastic strain fields in cross section XOZ with varying ratios between center distance of adjacent SiC fibers *d_0_* and fiber diameter *d_f_*: (**a**) *d*_0_*/d_f_* = 8, (**b**) *d*_0_*/d_f_* = 6, (**c**) *d*_0_*/d_f_* = 4, (**d**) *d*_0_*/d_f_* = 2.

**Figure 23:**
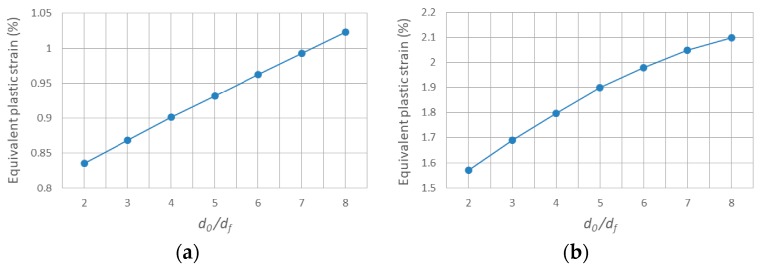
Plot of (**a**) the maximum equivalent plastic strain of residual indentation and (**b**) the equivalent plastic strain of point A under various ratios of *d_0_/d_f_*.

The equivalent plastic strain fields in cross section XOZ with varying ratios between the center distance of adjacent SiC fibers *d*_0_ and fiber diameter *d_f_* is shown in Figure 22. Note that the PEEQ in the center of the interface between the Ti and Al_3_Ti layer (point B) is barely affected by changing the ratio, which means the analysis focuses on the maximum PEEQ around the central SiC fiber (at point A, the closest location to upper boundary of the fiber) and of the residual indentation. Comparing the contours plotted in Figure 22a–d, note that, as the ratio of *d*_0_*/d_f_* goes down, less and less plastic strain permeates to the deeper layer of the material. Indicating that, when the SiC fibers are distributed more densely, the effect of fiber reinforcement on compressive strength is more visible. These observations can also be viewed from another scenario, which is shown in Figure 23a,b. The maximum PEEQ of residual indentation and the PEEQ of point A both increase with the growth of the ratio *d_0_/d_f_*. Implying that the densely distributed SiC fibers can effectively suppress the extension of plastic deformation, the strength of the Ti-(SiC_f_/Al_3_Ti) composite is improved when the center distance of SiC fibers get closer. In addition, according to Figure 23, the maximum PEEQ of residual indentation rises by 22.5% while the PEEQ of point A rises by 33.8% with the ratio of *d_0_/d_f_* ranging from 2 to 8. This suggests that the PEEQ of point A is more vulnerable for the change of center distance between adjacent fibers than the maximum PEEQ of residual indentation. Accordingly, it can be inferred that the sparsely distributed SiC fibers would cause higher plastic strain concentration at point A, which may lead to an earlier material failure around the fibers.

**Figure 24:**
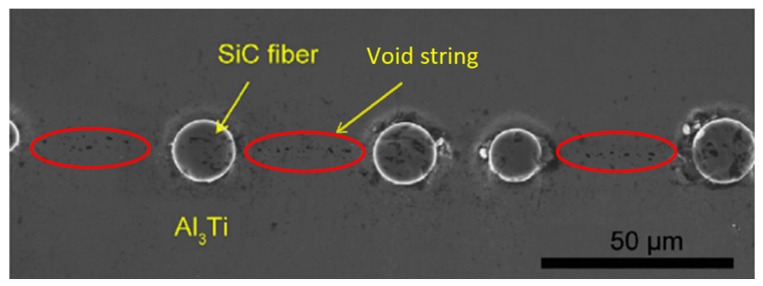
SEM image of SiC fibers in Ti-(SiC_f_/Al_3_Ti) composite showing the microstructure of void strings along the center line of SiC fiber string between adjacent fibers.

However, when the center distance gets too short, the plastic strain concentration induced by the stiff SiC fiber, which concentrated around the fiber before, extends along the horizontal center line of fiber string This is shown in Figure 22d. Due to the solid-state reaction during hot pressing process of the Ti-(SiC_f_/Al_3_Ti) composite [98], the material in the horizontal middle layer of the Al_3_Ti matrix is not intact and continuous, but a bonding layer includes many voids. As shown in Figure 24, it can be observed that there are void strings along the horizontal center line of the SiC fiber string between adjacent fibers. Therefore, the strength of this layer is weakened dramatically. So theoretically, the material can be identified as failing when the plastic strain concentration comes into being in this layer [96,99]. Accordingly, the optimal ratio between the center distance of adjacent SiC fibers *d*_0_ and fiber diameter *d_f_* is 4 in which the plastic strain ratio of the global material and the local position in the Al_3_Ti layer both drop significantly while the plastic strain concentration along the horizontal center line of the SiC fiber string between adjacent fibers has not yet come into being.

#### 5.4.2. Effect of Ti Volume Fraction

The ductile Ti layers provided sufficient bridging tractions to enhance fatigue resistance by almost an order of magnitude over the monolithic intermetallic known as Al_3_Ti [100]. Such a buffer effect on the load is closely related to the volume fraction of the Ti layer [101]. Therefore, based on the SAM model for the Ti-(SiC_f_/Al_3_Ti) composite in Section 4.1, the plastic strain fields in a half-space Ti-(SiC_f_/Al_3_Ti) composite with various volume fractions of the Ti layer are analyzed in this section in order to determine the optimal value for uncovering the best mechanical properties. The magnitude of load is fixed at 21.6 kN. The Ti layer with high ductility and toughness in a “Ti-Al_3_Ti-SiC-Al_3_Ti” unit is still considered an elastomer for simplicity and the influence on plasticity in the Al_3_Ti layer is of the greatest concern. The SiC fiber strings in the cases of this section are always located in the center layer of the Al_3_Ti layer.

**Figure 25:**
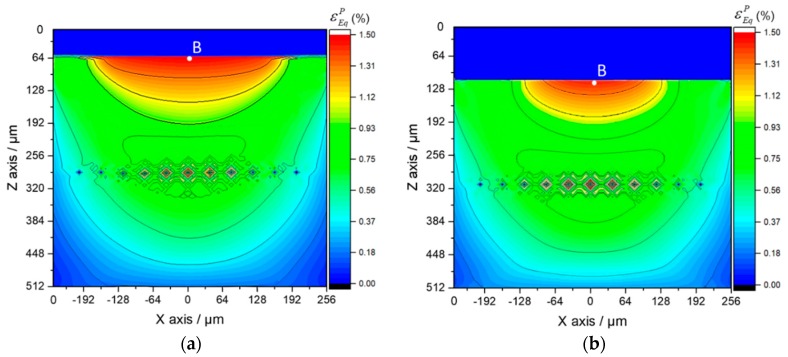

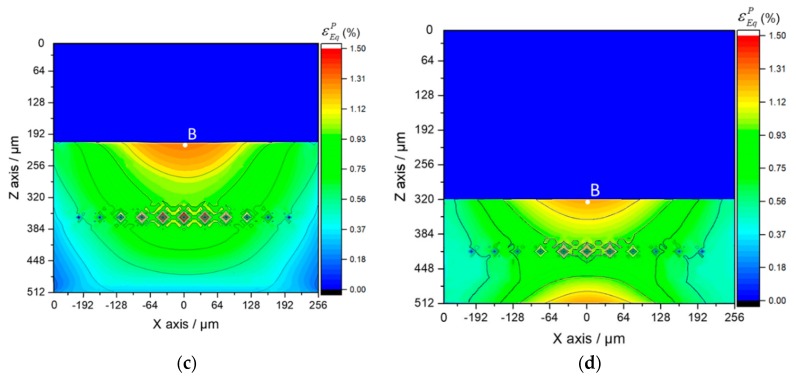
Equivalent plastic strain fields in cross section XOZ with varying volume fractions of Ti: (**a**) 10%, (**b**) 20%, (**c**) 40%, and (**d**) 60%.

**Figure 26:**
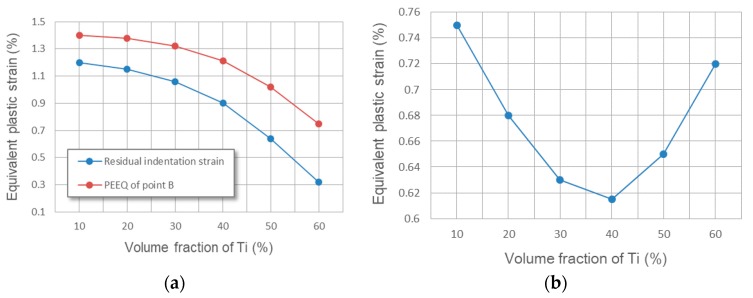
Plot of (**a**) the maximum plastic strain of residual indentation and the equivalent plastic strain of point B and (**b**) the average equivalent plastic strain of the Al_3_Ti layer with different volume fractions of Ti.

The equivalent plastic strain fields in cross section XOZ with varying volume fractions of Ti is shown in Figure 25. Considering that the variation in volume fraction of Ti affects the global plastic strain field much more dramatically than local fields, the analysis focuses on the maximum PEEQ at the interface between Ti and Al_3_Ti layers (at point B) and the residual indentation, which can better reveal the plasticity behaviors of the global field. Comparing Figure 25a–d, it can be observed that the plastic strain concentration region at the interface between two layers reduces both size and magnitude since the increasing thickness of the compliant Ti layer can provide a better buffer effect to the contact load. This indicated that a thicker layer of Ti do make a difference in improving the ductility and toughness of the Ti-(SiC_f_/Al_3_Ti) CCFR-MIL composite by effectively decreasing the plastic deformation at the interface of the Al_3_Ti layer. These observations can also be viewed from another scenario, which is shown in Figure 26a. The maximum PEEQ at the interface between two layers (at point B) and the residual indentation both drop with the growth of the Ti volume fraction by 46.4% and 73.4%, respectively. Implying that the maximum PEEQ of residual indentation is more vulnerable for the change of the Ti volume fraction than that at the interface between two layers. This is due to the increasing thickness of the elastic Ti layer, which can provide more resilience to the “Ti-Al_3_Ti-SiC-Al_3_Ti” unit. It also indicated that a thicker layer of Ti contributes more to improve the resilience than the ductility and toughness of the Ti-(SiC_f_/Al_3_Ti) CCFR-MIL composite.

However, the bending strength of the whole “Ti-Al_3_Ti-SiC-Al_3_Ti” unit is weakened as the thickness of the Al_3_Ti layer gets thinner. It can be observed from Figure 25d that the plastic strain concentration due to the bending of the whole unit has come into being at the bottom of the layer and the bending effect is highly possible. It amplifies the plastic strain concentration at the center of interface between Ti and Al_3_Ti layers. This shows that the plastic deformation of the whole composite material has expanded to a considerable degree. To quantitatively analyze the global plasticity behaviors, the definition of the average equivalent plastic strain is introduced [31]. As shown in Figure 25b, the average equivalent plastic strain of the Al_3_Ti layer declines with the growth of the Ti volume fraction before 40%. On the contrary, the value rises quickly when the volume fraction is over 40%. This implies that the plastic deformation degree of the whole composite material is at the lowest when the Ti volume fraction is 40%. Accordingly, the optimal volume fraction of Ti is 40% at which value the compliant Ti layer can provide a better buffer effect to the contact load without weakening the strength of the whole unit too much. This corresponds to the trend of the fatigue threshold [100] and stress intensity [101] with the increase of Ti volume fraction in the former references, which also fluctuate. Note that, the optimal volume fraction of Ti is around 20% to 30%, according to the references. This is because the CCFR-MIL composite in this paper is reinforced by the stiff SiC fibers in the Al_3_Ti layer while the MIL composite in references is not reinforced. As a consequence, the CCFR-MIL composite can allow a thicker compliant Ti layer existing in one “Ti-Al_3_Ti-SiC-Al_3_Ti” unit while reaching the same strength with the MIL composite.

## 6. Conclusions

A semi-analytical model for the sphere-plane elasto-plastic contact of the Ti-(SiC_f_/Al_3_Ti) CCFR-MIL composite based on the high-efficient numerical equivalent inclusion method is proposed to analyze the influences of the SiC fiber and Ti layer on the plasticity of the Al_3_Ti matrix layer. The investigation focused on microscopic strengthening, penetration, and failure mechanisms at the early stage of penetration when the laminated material along the loading direction was not damaged and the material was continuous. The parameters of magnitude of load, distribution of SiC fibers, and volume fraction of Ti were investigated for their effects on the plastic strain fields within the material measured by the equivalent plastic strains. In this way, the microstructure design of the Ti-(SiC_f_/Al_3_Ti) CCFR-MIL composite was optimized for better performance. The results indicated following major findings:

- In a single “Ti-Al_3_Ti-SiC-Al_3_Ti” unit after the hot pressing process, the thicknesses of Ti and Al_3_Ti layers are 216 μm and 296 μm, respectively. SiC fibers distribute in the horizontal center layer of the Al_3_Ti intermetallic layers and react with the intermetallic during the heat-treating process.
- The compressive strength and failure strain of the CCFR-MIL composite under sphere-plane contact load are ~1051 MPa and 3.01%, respectively. Both are lower than the values under the plane-plane contact load. In addition, the SiC fiber reinforcing effect for strength is still apparent while the effect for ductility is not as clear as that under the plane-plane contact load.
- Under the sphere-plane contact loading, the maximum plastic strain concentration in the Al_3_Ti layer is closest to the upper boundary of the central SiC fiber. It then extends along depth as the load increases, which are the locations where cracks may initiate and extend from.
- The optimal ratio between center distance of adjacent SiC fibers *d_0_* and fiber diameter *d_f_* under sphere-plane contact loading is 4 at which ratio the plastic strain of the global material and the local position in Al_3_Ti layer both drop significantly. When this occurs, the plastic strain concentration along the horizontal center line of the SiC fiber string between adjacent fibers has not yet come into being.
- The optimal volume fraction of Ti under sphere-plane contact loading is 40% at which fraction the compliant Ti layer can provide a better buffer effect to the contact load without weakening the strength of the whole material significantly.

## Figures and Tables

**Table 1:** Material properties.

Item	Values		
	Matrix	Hard Inhomogeneity	Soft Inhomogeneity
Young’s Modulus	200 Gpa	400 Gpa	100 Gpa
Poisson’s Ratio	0.3	0.3	0.3
Yield Strength	1000 Mpa	∞	800 Mpa
Isotropic Hardening Parameter	0.1	none	0.1

**Table 2:** Loading Conditions and model parameters.

Item	Values
Radius of Indenter	6 mm
Concentrated Load	50 N
Hertzian Contact Radius	0.1 mm
Hertzian Contact Pressure	2350 MPa
Size of Solution Domain for SAM (axisymmetric model, *x*, *y*, *z*)	0.64 mm × 0.64 mm
Size of Solution Domain for SAM (3-D model, *x*, *y*, *z*)	0.256 mm × 0.256 mm × 0.256 mm
Element Size of FEM Model (local refinement area)	1 μm
Element Size of SAM Model	4 μm
Radius of Cylindrical Inhomogeneity	0.064 mm
Generatrix of Cylindrical Inhomogeneity	0.064 mm
Depth of Inhomogeneity Center Beneath Contact Point	0.064 mm

**Table 3:** Parameters of the new NEIM-based SAM model in validation.

Terms	Al_3_Ti Layer	SiC Fiber	Ti Layer
Mesh Size, SAM, (μm)	8		
Element Number, SAM (*x*, *y*, *z*)	64, 64, 64		
Radius of Indenter (mm)	4		
Thickness of Inhomogeneity Ti layer *h_Ti_*, (μm)	216		
Thickness of Matrix Al_3_Ti layer *h_Al_3_Ti_*, (μm)	296		
Maximum Load of Indenter, (kN)	21.6		
Depth of SiC Inhomogeneity Horizontal Center Line *h_i_*, (μm)	/	364	/
Radius of SiC Inhomogeneity *r_0_*, (μm)	/	4	/
Distance between SiC Inhomogeneity Centers, *d_0_*, (μm)	/	8*r_0_*	/
Ratio of Young’s Modulus *E*/*E_Matrix_*	1	1.11	0.63
Poisson’s Ratio	0.28	0.3	0.34
Initial Yield Strength (MPa)	1003	841	/
Hardening Parameter	0.1	0.1	/

**Table 4:** Compressive properties of Ti-(SiC_f_/Al_3_Ti) CCFR-MIL and Ti/Al_3_Ti MIL composite under different loading conditions.

Materials	Contact Mode of Loading	Compressive Strength (MPa)	Failure Strain (%)
Ti/Al_3_Ti	Plane-plane	1332	3.34
	Sphere-plane	987	3.19
SiC-Ti/Al_3_Ti	Plane-plane	1453	3.30
	Sphere-plane	1051	3.01
